# Scoping review of the impacts of urban agriculture on the determinants of health

**DOI:** 10.1186/s12889-019-6885-z

**Published:** 2019-05-31

**Authors:** Pierre Paul Audate, Melissa A. Fernandez, Geneviève Cloutier, Alexandre Lebel

**Affiliations:** 1 Graduate School of Land Management and Regional Planning, Faculty of Planning, Architecture, Art and Design, Laval University, Quebec, G1V 0A6 Canada; 2 Centre for Research on Planning and Development (CRAD), Laval University, Quebec, G1V 0A6 Canada; 3 Institute of Nutrition and Functional Foods, Laval University, Quebec, G1V 0A6 Canada; 4 School of Nutrition, Faculty of Agricultural and Food Sciences, Laval University, Quebec, G1V 0A6 Canada; 5 Evaluation Platform on Obesity Prevention, Quebec Heart and Lung Institute, Quebec, G1V 4G5 Canada

**Keywords:** Urban agriculture (UA), Determinants of health, Food, Health, Scoping review

## Abstract

**Background:**

There has been an increasing interest in urban agriculture (UA) practice and research in recent years. Scholars have already reported numerous beneficial and potential adverse impacts of UA on health-related outcomes. This scoping review aims to explore these impacts and identify knowledge gaps for future UA studies.

**Methods:**

A systematic search was conducted in seven electronic bibliographic databases to identify relevant peer-reviewed studies. Articles were screened and assessed for eligibility. From eligible studies, data were extracted to summarize, collate, appraise the quality and make a narrative account of the findings.

**Results:**

A total of 101 articles (51 quantitative, 29 qualitative, and 21 mixed methods studies) were included in our final analysis. Among these articles, 38 and 37% reported findings from North America and Sub-Saharan Africa respectively. Quantitative studies revealed evidence of positive impacts of UA on food security, nutrition outcomes, physical and mental health outcomes, and social capital. The qualitative studies reported a wide range of perceived benefits and motivations of UA. The most frequently reported benefits include contributions to social capital, food security, health and/or wellbeing. However, the evidence must be interpreted with caution since the quality of most of the studies was assessed as weak to moderate. While no definitive conclusions can be drawn about the adverse impacts of UA on health, paying particular attention to contamination of UA soil is recommended.

**Conclusion:**

More peer-reviewed studies are needed in areas where UA is practiced such as Latin America and Caribbean. The inconsistency and the lack of strong quality in the methodology of the included studies are proof that more rigorous studies are also needed in future research. Nevertheless, the substantial existing evidence from this review corroborate that UA can influence different determinants of health such as food security, social capital, health and well-being in a variety of contexts.

**Electronic supplementary material:**

The online version of this article (10.1186/s12889-019-6885-z) contains supplementary material, which is available to authorized users.

## Background

Until recently, food systems were given little attention in the agenda of urban planners [1]. Urban agriculture (UA) is an example of food system components with little or no existing regulations in many cities worldwide. In the last decades, practitioners have been advocating for the inclusion of UA in urban planning policies [2]. This has opened new avenues for research on UA in a wide range of disciplines.

Numerous beneficial and potential adverse impacts of UA have been reported in urban planning and public health fields [3, 4]. Studies on urban gardens in high-, middle-, and low-income countries suggest they influence several food security and nutrition outcomes [5, 6]. For example, in the United States (US), participation in community gardening (a type of UA intervention when it is practised in urban settings) increased fruit and vegetable (F&V) consumption of gardeners in comparison to their non- gardening counterparts [7, 8]. Greater F&V consumption is associated with health improvements and prevention of chronic diseases [9]. UA related activities have also demonstrated an influence on physical and mental health outcomes.

A study conducted in two large community garden networks in Salt Lake City, Utah has demonstrated that UA is a good physical activity that can prevent obesity. This study revealed that the community gardener participants had significantly lower body mass index (BMI) compared with their neighbors who did not participate in community gardening activities [10]. The positive role of urban gardening in human well-being has also been explored [11]. Additionally, urban gardening has been proven to positively influence stress reduction outcomes [12], foster social cohesion while providing participants the opportunity to build social networks and connect to their community [13].

Despite these potential positive effects on a variety of health determinants, researchers are demanding for further clarity on the benefits of UA [14]. Adverse impacts of UA have also been reported by the public health community and urban planners. Several studies showed UA practices can influence food safety because of the risks associated to urban soil or water contamination [15, 16]. Other studies have pointed out the facts that urban gardening can be a place where certain participants feel excluded or it can also be a place where existing race and social class-based disparities are replicated [17]. All these assumptions and evidence make the literature on UA impacts on health outcomes very diverse.

The diversity of evidence in the literature could be explained by different methodological approaches, a focus on a specific aspect of UA, or the socioeconomic context where UA is implemented. This scattered knowledge makes it difficult to help urban planning stakeholders and could possibly misguide decision making; and would benefit from a synthesis of scientific knowledge on this matter.

To our knowledge, there is only a limited number of systematic reviews on this topic [18–21]. While three literature reviews [18, 19, 21] have focused on the beneficial impacts of UA on specific food security or nutrition outcomes such as dietary intake, nutritional status, or healthy food access, they have not considered potential adverse impacts. Guitart et al. [20], has taken a broader approach to synthetize the existing knowledge by also including the adverse impacts. However, this review only considered urban community gardening which is a specific type of UA that does not include other types such as backyards, domestic gardening, or individual owned farms.

Furthermore, beyond how UA was defined by authors, reviews showed a lack of diversity in the socioeconomic context and geographic scope in included primary studies. While Poulsen et al. [19] and Warren et al. [18] mainly included studies from low- and middle-income countries from Sub-Saharan Africa’s region, most of the primary studies included by Guitart et al. [20] were from the US, a high-income country. Only one primary study [22] from Sub-Saharan Africa’s region was included into the final analysis of Guitart et al. [20]‘s study. While Poulsen et al. [19] only explored low-income countries, in Warren et al. [18], socioeconomic contexts were not an exclusion criterion. Three primary studies from high- income countries identified [7, 8, 23] were purposely excluded from Warren et al. [18] final analysis because the number was considered too low in terms of studies to include.

Based on these observations, there is still a need for systematic reviews that explore the impacts of UA in a broad socioeconomic context and geographic scope. By synthesizing vast amounts of literature, a systematic review can provide insights into understanding the general or common characteristics of individuals and communities involved in UA and how this activity affects their health.

For this paper, the determinants of health are personal, socioeconomic, environmental and cultural factors that influence a person’s or community’s health. They include lifestyle, food, social and community networks, sanitation, environment etc. [24].

The aim of this study was to explore the impacts of UA on the determinants of health and identify knowledge gaps for future UA studies by conducting a scoping review of peer-reviewed literature. The following research questions were investigated: i) what are the impacts of UA on the determinants of health? and ii) how do these impacts differ according to countries’ income level (high-, middle-, and low-income) and sociodemographic characteristics of participants? The responses to these questions will allow us to present the geographical location of UA studies, the type of impacts (positive or adverse) studied, and the methods utilized by scholars to assess the impacts of UA on the determinants of health.

## Methods

A systematic literature review on the impacts of UA on health determinants was performed. The wide range of health determinants, methods and results used in UA research suggests the use of a scoping review as described by Arksey and O’Malley [25] and Levac et al. [26]. A scoping study adopts a broader search strategy while allowing reproducibility, transparency, and reliability on the current state of literature. The detailed protocol of this scoping study that includes the search strategy and steps of the systematic review process has been published elsewhere [27]. Briefly, the search strategy included a set of keywords on UA, and determinants of health identified with the help of a library specialist for electronic bibliographic search. An additional file shows the keywords in detail (see Additional file 1).

### Identification of relevant studies

Original peer-reviewed articles published in English language journals from January 1980 to December 2017 were obtained from systematic searches of seven electronic bibliographic databases that include: PubMed, Embase, MEDLINE (Embase), CINAHL Plus with full text, Academic search premier (EBSCO host), CAB Abstract (ovid), and Web of science in January 2018. The final search strategy for PubMed can be found in an additional file (see Additional file 1). All identified articles from the searches were transferred to a reference manager software (EndNote, X8 Thomson Reuters) and all duplicates and titles in other languages were removed. The EndNote (X8 Thomson Reuters) file was later transferred to an online systematic review software (Distiller SR, Evidence Partners, Ottawa, Canada) for screenings. The PICOS (participants, intervention, context, outcomes, and study design) framework [28] was used to establish eligibility criteria.

In order to be included, original peer-reviewed articles had to meet five criteria. First, the study considered UA as a food growing initiative that involves participants. Soil and water contamination studies that did not specifically assess risks for humans were excluded. Second, the focus of the study was UA defined as a food growing initiative in urban settings. Studies that combined other interventions with food production (e.g. school gardening programs that include cooking lessons [29–31]) were excluded due to our inability to ascertain the independent effect of UA on the targeted health outcome. Third, the study was conducted in urban areas. All studies that explicitly stated they consider rural, peri-urban, or suburban areas were excluded unless the results were desegregated to make comparisons with urban areas. Fourth, at least one of the outcomes measured or findings reported in the study were determinants of health as listed in Table 2. Fifth, only peer-reviewed articles written in English that describe original quantitative, qualitative, or mixed methods research were considered. Grey literature, narratives, commentaries, or other document types such as reports, and essays were excluded. Systematic reviews were also excluded; however, the reference lists of all eligible ones were carefully revised for additional relevant studies.

### Selection of relevant and reliable studies

By applying the eligibility criteria, two reviewers (PPA with background in agriculture and MAF with background in nutrition) have screened the articles for selection. The first selection was from title and abstract screening and the second one was from a full-text screening. All conflicts generated through the screening stages between the two reviewers were discussed until consensus was reached. When needed, a third opinion from two other authors (AL and GC) was consulted to reach consensus.

### Data extraction from included studies

Once the articles were selected, the following data were recorded in a spreadsheet: author(s), year, city, region, country’s income level, level of influence (e.g. individual, household or community), characteristic of participants (e.g. children, adults), type of UA (e.g. community gardening, home gardening, allotment, school gardening, and urban farming), study purpose, study design (e.g. quantitative, qualitative, or mixed methods), measurement methods, outcomes measured, and key findings. One author extracted the data, and another validated them to ensure accuracy prior the quality appraisal phase.

### Study quality appraisal

For the quality appraisal of the included articles a checklist (see Additional files 2 and 3) was developed using Wallace et al. [127] criteria and a modified rating system as suggested by Ohly et al. [128] for the qualitative studies. Given the mix of study methods found in the quantitative studies (cross-sectional, randomized controlled trials, before and after surveys, risk assessment), it was not appropriate to consider only one existing quality assessment tool to appraise the quality of quantitative studies. The authors have instead opted to develop a 12-item checklist based on criteria and questions from the following three quality assessment tools sources: i) assessment tool for observational cohort and cross-sectional studies, and assessment tool for before-after studies with no control groups [129], ii) quality assessment tool for quantitative studies from the Effective Public Health Practice Project (EPHPP), and iii) study limitations and ethical criteria [127]. We used the same overall rating system for quantitative and qualitative studies. The first author (PPA) appraised the quality of the included studies and obtained validation from the second author (MAF). When needed, a third opinion from the other two authors (AL and GC) was consulted.

### Collating, summarizing and reporting the findings

A narrative account of the included studies was prepared to present patterns in UA impacts on the determinants of health. A numerical analysis presented the number, geographical distribution, and type of UA of the included studies. Since the outcomes were broad, they were synthetized thematically to record the overall impacts of UA as positive, adverse, neutral, or mixed for the quantitative or mixed methods studies in some cases. The neutral impact was assigned to studies that presented quantitative measurement tools but did not present significant results as positive or adverse effect of the measured outcomes in their findings. The mixed impact was used to classify studies that presented both positive and adverse effects of the measured outcomes. On the other hand, the terms perceived benefits, challenges or motivations were used to classify the outcomes of the qualitative and the remaining mixed methods studies. The reported outcomes and findings were synthetized and grouped into specific themes defined by the authors to alleviate the narrative account (Table 2).

## Results

### Identification of potential studies

**Fig. 1:**
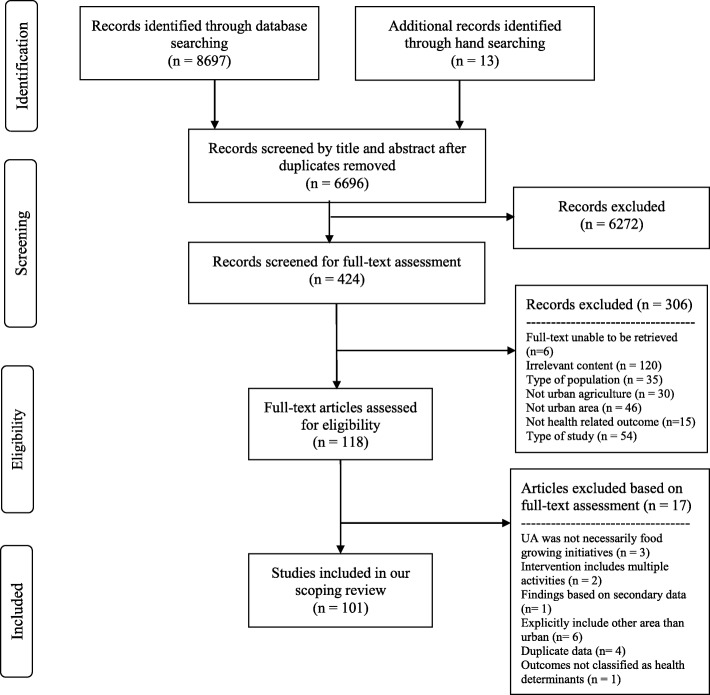
Flow chart of the studies identification and selection process

The searches from the seven electronic databases hit a total of 8697 records (Pubmed: 674, Embase: 791, Medline: 637, CINAHL Fulltext: 295, Academic search premier: 692, CAB abstract: 2506, Web of science: 3102) that led to a total of 6683 titles and abstracts that were screened after the removal of duplicates. We retrieved a total of 418 full-text articles from our different libraries. Six records were unable to be obtained in full-text format. The full-text screening’s stage led to 118 potential articles relevant to our scoping review. Additional articles were excluded after full-text assessment for the reasons mentioned in the flowchart (Fig. 1). A total of 101 articles were therefore included in our final data extraction, quality appraisal, and narrative account stages.

### Characteristics of the included studies

The peer-reviewed literature on the impacts of UA on the determinants of health is recent and it has considerably increased in the last few years (Table 1). Among the included studies, 61% were published in the last five years of this current study (2013–2017) and approximately, 90% have been published in the last decade (2007–2017) of this current study.

**Table 1:** Number of included articles by decade (1980–2017)

Year	Number of studies
1980–1990	0
1991–2000	2
2001–2010	19
2011–2017	80
Total	101

**Fig. 2:**
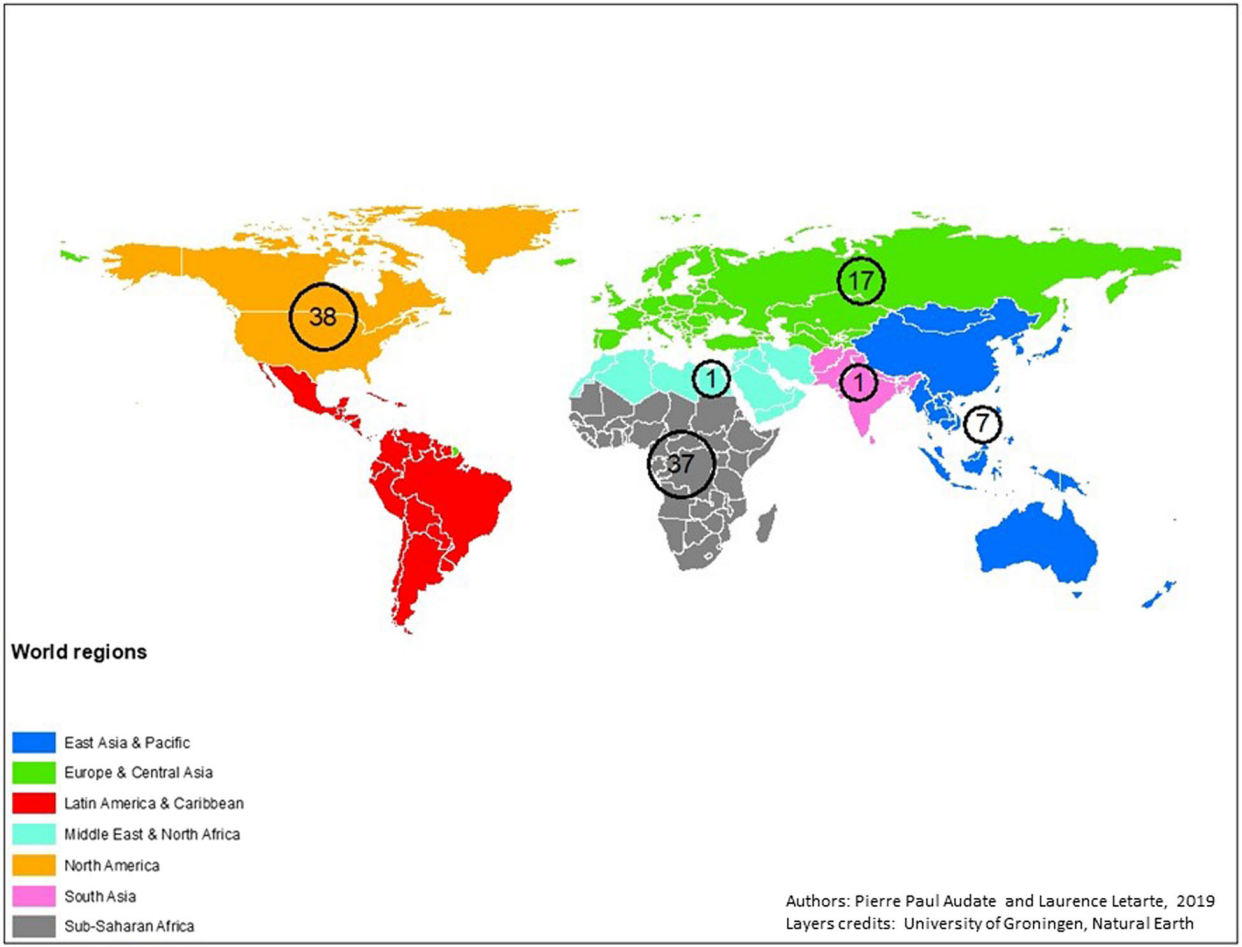
Number of included studies by world regions

In terms of geographic scope of the included studies, they are mainly from two world regions where 38 and 37% were conducted and reported findings from North America and Sub-Saharan Africa respectively (Fig. 2). Research in North America was predominantly from the US which alone has 33 of the 101 included studies. In the case of Sub-Saharan Africa’s region, the studies are divided among several countries. For example, the country with the highest number of included studies in this region is Nigeria with a total of nine studies. In addition, at least 12 other countries from this region are represented in our list of included studies.

Out of the 101 included studies, 59% were focused on high- income countries, 32% in middle- income, 8% in low- income and 1% in both (middle- and low- income) countries. In addition, there is a diversity of countries *n* = 34 in total where the impacts of UA on health-related outcomes have been studied.

### Type of methods and design

The included studies in our research have used three types of study design: *n* = 51 used quantitative methods, *n* = 29 used qualitative methods, and *n* = 21 have explored mixed methods (Table 2). Among the quantitative studies *n* = 14 are health assessments, *n* = 25 used cross-sectional surveys, n = 2 used both health assessment and cross-sectional surveys, *n* = 4 quasi-experimental designs, n = 1 randomized control trial, n = 1 before and after or pre and post surveys, and n = 4 case studies. The qualitative and mixed methods used a wide range of measurement methods to collect data such as in-depth and semi-structured interviews, focus groups, surveys, and observation questionnaires (see Additional file 4). They have also used a wide variety of qualitative approaches that include ethnography, grounded theory, and case studies. However, in most of the cases, it was difficult to identify the qualitative approaches because the authors did not provide enough details on their methodology.

**Table 2:** Characteristics of health-related outcomes assessed by the included studies

Reference and study design						
Determinants of health	N	Quantitative	Qualitative			Mixed methods
			Perceived benefits	Perceived challenges	UA Motivations	
Food security	47	[7, 32–50]	[51–53], [54]^a^, [55–57], [58–60]^b^, [61, 62]		[54]^a^, [63]	[64–77]
Social and community networks (social capital)	36	[33, 45, 48, 78], [79]^b^, [80–82]	[52, 55], [58]^b^, [59]^a,b^, [60]^b^, [61, 62], [83], [84]^b^, [85–91], [92, 93]^b^	[51, 56] [59]^a^,^b^	[63]	[66, 68, 73, 75, 94–96]
Health and/or well-being	24	[10, 33, 43, 45, 78, 82], [97, 98]^b^	[5, 51, 55, 89, 90], [92]^b^, [99], [100]^b^, [101], [102]^a^		[102]^a^	[67, 68, 73, 74, 94, 103]
Sanitation and/or food safety	24	[15, 16, 37, 104–119]		[5, 55]		[77, 120, 121]
Income, cost savings and/or employment	23	[32, 34, 36, 41–43, 45, 47, 122]	[56, 57], [58]^b^, [83], [100]^b^		[101]	[66, 67, 69, 71–73, 76, 95]
Nutrition	17	[7, 8, 33, 40, 44, 47–49, 80], [123]^b^, [124]	[5], [92]^b^, [101]			[70, 103, 125]
Natural and/or physical environments	9	[80, 126]	[83], [84]^b^, [102]	[5], [57], [92]^b^		[72]
Cultural connection	8		[52, 53, 61, 86, 88]		[63, 91]	[77]
Lifestyle	6	[37, 78], [98]^b^	[84]^b^		[91]	[65]
Education and/or empowerment	5	[44]	[60]^b^, [90]			[103, 125]

^a^Findings were discussed in more than one category

^b^Study quality was rated as strong

### Quality appraisal of the included studies

All types of included studies were assessed for the quality of the outcomes and findings reported. Those which quality was appraised as strong are identified in Table 2. The quality of quantitative and qualitative aspects of mixed-methods studies was appraised separately (see Additional files 2 and 3). Overall, the majority of studies reporting quantitative data were appraised with weak or moderate quality ratings. Only four quantitative studies were rated as strong. Most of the studies that scored weak or moderate did not provide enough information and details to justify their population size and used cross-sectional study designs without repeated measurements or control groups. More than half of them did not address limitations and ethical issues related to their study design. Similarly, more than 90% of the studies that reported qualitative data were also rated as weak or moderate. Only, seven qualitative studies were rated as strong studies. The majority scored moderate or weak because they do not provide enough information on their data collection, theoretical approach, methods, and did not address limitations or ethical issues (see Additional file 3).

### Type of UA studied

The included articles used a variety of terminology to study UA. Among the most commonly type of terminology used: *n* = 36 partly or entirely explored community gardening, *n* = 19 studied urban or commercial farming, *n* = 9 explored home or backyard gardening, *n* = 7 used the term allotment gardening, n = 7 were focused on institutional type of UA such as school gardening, church gardening, or gardening on university campuses. Urban livestock, urban rooftop farming, sack gardening, are also among other terms used to identify UA activities (see Additional file 4).

### Type of health-related outcomes assessed

The quantitative outcomes assessed and qualitative themes that emerged were grouped into ten categories inspired from the determinants of health model [24] (Table 2). Most studies investigated multiple determinants of health such as food security, nutrition, social capital. Among the studies that measured food security outcomes, 7 (5 quantitative, 1 qualitative, and 1 mixed methods study) reported findings only on food security outcomes. Among the ones focused on nutrition, there are three quantitative studies that assess only nutrition outcomes (see Additional file 4).

### Quantitative studies

#### Food security and nutrition outcomes

Among the studies that investigated food security outcomes 75% reported findings that demonstrated the positive impacts of UA on food security. Two studies [42, 43] reported findings that influenced participants both positively and negatively. Three studies [36, 39, 47] were neutral because they did not provide evidence of any impacts on food security.

Eleven quantitative studies investigated nutrition outcomes (Table 2). Among them, UA was reported to positively influence F&V intake of participants in five studies [7, 8, 33, 44, 80], nutritional status of children in two studies [49, 124], and food diversity in one study [40]. Two studies [47, 123] did not provide any evidence of impacts of UA activities on nutrition outcomes. For example, Christian et al. [123] used a strong quantitative study design to measure F&V intake among children that do school gardening activities. However, its findings failed to support that school gardening improves children’s daily F&V intake.

#### Social capital

Eight quantitative studies explored social capital (Table 2). All of them have reported positive impacts or benefits of UA activities on social capital. Soga et al. [82] used a Social Cohesion and Trust Scale to statistically demonstrate that gardeners have greater social cohesion than non-gardeners. Litt et al. [80] reported on the social capital by exploring outcomes such as social involvement or collective efficacy of gardeners and the study concludes that urban gardeners have more involvement in social activities than non-gardeners. Based on the findings from the other studies, we can claim that UA gardeners have higher social support than non-gardeners [78]. UA can also positively influence friendship and adaptability between friends [79] or different ethnic groups [81].

#### Health and/or wellbeing

Among the studies that reported findings and outcomes related to health and/or wellbeing, some reported positive impacts of UA on physical health in general [33, 78] or physical health-related outcomes such as BMI and obesity risk [10] and improved muscle mass [98]. But UA activities do not always influence positively BMI as three studies [33, 78, 82] did not find significant positive impacts of UA on BMI. Other studies reported outcomes that were related to the health of people with mental disabilities [97] or mental health [82]. Three studies [45, 78, 98] also reported well-being as UA benefits. For example, Park et al. [98] found that UA activities improve psychological health of women by demonstrating that women participants of UA activities exhibit lower depression score compared to their control groups. Hawkins et al. [78] reported significant difference in perceived stress levels between allotment gardeners and other participants of indoor activities. One study [43] mentioned some health problems such as headache related to UA activities.

#### Sanitation and food safety

Among the quantitative studies that addressed issues related to health concerns or food safety, one [37] positioned food safety as one of the most important motivations for UA practitioners. Three studies [104, 109, 110] that assessed health risk due to heavy metal contamination were neutral because they found that the contamination of the soil or produce pose no risk to human groups assessed. The remains reported potential adverse impacts of UA. Matthys et al. [111] and Stoler et al. [117] found significant associations between UA activities and the risk of malaria among urban farming households in Sub-Saharan Africa’s region. Antwi-Agyei et al. [105] found that use of wastewater in UA can expose farmers in Africa to pathogenic agents such as *E. coli*. Grace et al. [108] studies urban livestock and found that children under five years in dairy households were exposed more to *Cryptosporidium oocysts*. Other authors assessed potential contamination of urban soil and UA produce by heavy metals. Most of them agreed that accidental ingestion of UA soil [106, 115, 116, 119] or consumption of vegetables or other produce grown in contaminated UA soil [15, 16, 106, 107, 112–114, 118] may represent a risk for the health of different population groups (e.g. children and/or adults).

#### Income and cost savings on food

Quantitative studies also reported findings on income, cost savings on food, and/or employment. UA was reported as an activity that provides income to farmers in the African context [32, 122], other studies preferred to relate UA as an activity that allow practitioners to save money on food expenses and this statement has been put into evidence in different world region such as North America [36, 47] or Sub-Saharan Africa [41]. A study conducted in the US by Algert et al. [34] states that UA allows gardeners to save $339.00 by growing their own vegetables. Other studies [42, 43, 45] have reported the income related findings in terms of motivations and perceived benefits of UA practitioners.

### Qualitative studies

#### Perceived benefits of UA

Out of 29 qualitative studies, 26 addressed several perceived benefits of UA for practitioners. The most commonly mentioned benefits include: contribution to food security and nutrition, in terms of access to fresh or healthier foods [51, 53, 92], enhanced health and wellbeing, foster social capital, strengthen cultural connections, education, savings on food expenses, and/or a source of income (Table 2).

#### Motivations on UA

The remaining three qualitative studies included mainly discussed the motivations of people involved in UA. Among the wide range of motivations expressed by people engaging in UA, the studies mentioned: food or savings on food expenses, opportunity to build social connections, environmental consciousness, stress reduction, leisure, and other health related reasons (e.g. healthier lifestyle and/or diet diversity).

#### Challenges related to UA

Seven studies discussed challenges related to UA (Table 2). Among the main challenges discussed: insecure land tenure, violence perception, and food safety concerns of community-garden participants, and social exclusion due to people who feel excluded in some community gardens are concerns that may require attention from UA stakeholders.

### Mixed methods studies

The evidence from mixed methods studies presents a set of UA impacts similar to those described in the previous sections for the quantitative and qualitative studies. However, the findings were dominated by qualitative evidence. Only six of the studies [64, 69, 71–73, 125] presented quantitative evidence in their findings. Panneerselvam et al. [73] and Mkwambisi et al. [71] presented findings that demonstrate UA activities positively influence food security outcomes. For example, in Malawi, low-income female-headed households consumed 34.3 and 11% of the total UA harvest. The UA impacts have also positively influenced savings on food. In India, 30% of the farmers experienced 20–40% reduction in food expenditure [73]. Mlozi [72] also reported positive impacts of UA activities on food security and income, arguing that the profits of urban farmers were seven times higher than a senior government’s official. However, it also addressed some concerns related to environmental damage of urban livestock. Miura et al. [70], who studies a set of nutrition and food security outcomes, was not able to conclude whether or not UA activities improved the diet of the participants. One study found that UA positively influenced social capital. For example, 87% of participating farmers agreed that relationship with their neighbours improved because of UA [73].

The remaining studies described a wide range of motivations, perceived benefits, and challenges of UA. Among the challenges documented is the fear due to potential food contamination and exposure of UA practitioners and their families to contaminants [77]. Gallaher et al. [120] and Kaiser et al. [121] assessed health risk perception due to UA activities in potential contaminated soil and found respectively that farmers and urban residents were aware and worried that potential hazards such as heavy metals could contaminate food grown in the gardens. Finally, other perceived burdens as barriers to participate in UA activities such as: hard work, getting dirty, and feeling unsafe [65] are also reported.

### Level of influence of the outcomes

**Fig. 3:**
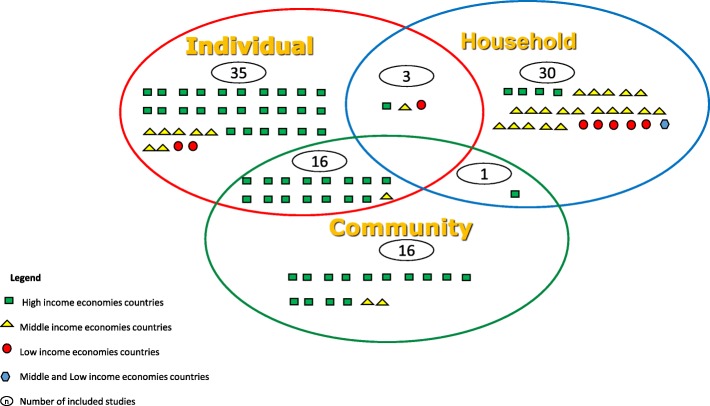
Number of included studies based on levels of influence of the impacts of UA on the determinants of health and country-income levels

The included studies were categorized into three different influence levels (individual, household, and community) to measure or demonstrate the influence of UA on the determinants of health. Most of the studies from high-income countries demonstrate or measure the impacts at individual or community levels. On the other hand, studies from middle- and low- income countries explored the impacts mostly at household and individual levels (Fig. 3).

## Discussion

This scoping review used standard systematic review methods to identify, select, and synthesize findings from 101 studies that reported impacts of UA on the determinants of health. We documented the state of UA peer-reviewed literature by analyzing the geographic scope, country-level income, type of UA activities, and key findings on the main reported determinants of health. Below, we provide important information on the implications of the findings and the gaps that emerged from the results of this review that can be relevant for UA practitioners, researchers, and policy makers.

The results from the included quantitative and mixed method studies revealed some substantial evidence on the positive impacts of UA on food security and nutrition outcomes with increasing F&V consumption, improving food security status of urban farmers or nutritional status of children, food diversity, and/or dietary intake. However, this evidence has to be interpreted with caution. The outcomes reported are mainly based on cross-sectional surveys that rely on the participants’ self-reported responses. Most studies did not use validated tools for food security and nutrition outcomes’ measurement. In addition, in most cases, the authors do not always provide rigorous statistical evidence to sustain their findings. Other studies [39, 47, 70, 123] were not able to find enough evidence that justify the positive impacts of UA on food security or nutrition outcomes.

Although social capital is a determinant of health with limited reliable and valid measurement tools [130], it is less common to find studies that only use quantitative methods to measure social capital. In this review, social capital was an important determinant of health where the positive impacts of UA have been strongly supported by quantitative studies [79, 82]. Nevertheless, some caution regarding methodological limitations (cross-sectional studies without repeated measurements, sample size justification) should be considered when interpreting these findings as more rigorous studies are needed to corroborate the evidence.

Several studies reported the adverse impacts of UA on health by assessing the risks related to consumption of food grown in contaminated urban soil. However, the findings do not allow to draw definitive conclusions on this topic. Most of the findings are based on authors’ assumptions of the amount of produce consumed or soil accidentally ingested by the population. This method is limited since it does not always reflect reality. In addition, in regard to ethics, it may be difficult to find the right way to assess health risks. This is because it is unethical for researchers to intentionally ask participants to consume contaminated produce in order to take the correct measurements. In order to improve the reliability of this type of data, it is probably better to record the real amount of produce consumed by the studied population.

The findings from qualitative studies highlight a wide range of perceived benefits and motivations of UA. The benefits reported by UA practitioners were similar to their motivations. Supplying food in adequate quantity or quality, building social capital, improving physical and mental health, and saving on food expenses were the most common reasons and benefits perceived by UA practitioners. Other less common but important reasons include income, heathy lifestyle, and education and environmental consciousness [58, 83, 90, 101]. Other benefits of UA activities such as personal development have already emerged from other systematic reviews [131]. On the other hand, each study showed findings from their specific context. But the results showed heterogeneity in the types of UA activities and diversity of the methods used. Unfortunately, we were not able to appreciate much difference between countries’ income level and the outcomes assessed.

In this case, most of the determinants of health’ themes emerged were explored in high-, middle-, and low- income countries. Lifestyle and cultural connection were the only two themes that appeared in high-income countries but did not in middle- or low- income countries. We expected some outcomes such as food security and nutrition to be associated more with middle- and low- income countries. However, they were also importantly assessed in various studies from high- income countries. This highlights a fact that other authors have already pointed out that food is also an important function of UA in the context of high-income countries [132].

We also found that scholars from high- income countries are more likely to study the impacts of UA at individuals and/or community levels while studies from middle- and low- income countries are more likely to explore the contributions of UA on determinants of health at household and individual levels without considering the community aspect. This trend can be explained by the fact that community gardening is a type of UA with more presence in high-income countries [20] compared to other low- and middle- income countries where other types of UA such as home gardening or urban farming are more common. In other words, the urban farming as a larger type of UA practiced in middle- and low- income countries, is more likely to engage the entire household unlike the community gardens where sometimes the plots are smaller and only one member of the household is involved.

Another important aspect that was observed from our review is the lack of transnational or multi-city studies. Only one included study, Frayne et al. [39], which published findings from the same data as Crush et al. [6], was conducted in more than one country. Only seven out of 101 included studies have been conducted in more than one city. These finding prove that despite the diversity in the geographic scope and types of UA of the existing academic literature, UA remains a topic studied in specific or local contexts and that partly limits the capacity to generalize its potential impacts on specific determinants of health.

Aside from the US and Sub-Saharan Africa, there is limited peer-reviewed research in other world regions where UA is highly recognized and practised. For example, we did not find eligible studies in the Latin American and Caribbean’s region. However, cities such as Belo Horizonte in Brazil, Havana in Cuba, Rosario in Argentina and Quito in Equator from this region have been widely recognized as successful UA cases for their urban and peri-urban food practice and policy [133]. Among the possible explanations for the lack of studies from other world regions are the dominance of the academic literature on UA by countries from North America and Sub-Saharan Africa, and the exclusion of peri-urban area in our definition of UA. In addition, our review only considers English language bibliographic databases and journals, which may have overlooked relevant studies published in other languages. However, since English is considered a hegemonic language in the international scientific literature [134], we also expected to retrieve more eligible papers published in English from other world regions where English is not the first language.

All types of studies (quantitative, qualitative, and mixed methods) were predominantly qualified as weak or moderate. The inconsistent or incomplete reporting of results from some included studies were due to lack of details on study settings, sample size justification, data collection, ethical issues, statistical evidence for quantitative studies, and theoretical approaches for qualitative studies. These arguments strongly support a lack of methodological rigor in the evidence of the impacts of UA peer-reviewed literature and add on the evidence already mentioned by several authors [18, 19, 21].

### Strengths and limitations of this scoping review

This review applied a systematic and rigorous search strategy that retrieves several articles to answer our research questions and objectives. As our topic was focused on UA and health, several well-known electronic bibliographic databases related to health, nutrition, and agriculture were used as primary sources. Each element from the PICOS framework was searched with multiple keywords in order to target all relevant studies [27]. However, we may have omitted some relevant studies published in other languages. Based on the geographic scope of the included studies, it is important to point out the existence of English language academic literature on the impacts of UA, but it is mostly focused on the US and some countries in Sub-Saharan Africa.

No study on air pollution and UA was included in our final analysis. This can be explained by the fact that we have unintentionally omitted air pollution as a key word in our search strategy. Additionally, we only considered peer-reviewed articles without assessing the existing evidence in the grey literature. The non-consideration of the grey literature restricts our findings to what was reported by scientific journals and possibly prevent the analysis of relevant cases that were rejected for publication by scientific editors.

### Study implications

Our study reveals a need for more rigorous studies to demonstrate the impacts of UA on health-related outcomes and the possibility of exploring more transnational and multi-city research approaches to enrich the understanding on different contexts. This will help document best practices that can be implemented across different settings and contexts. As we stated earlier, UA remains a topic studied in specific or local contexts and that partly limits the capacity to generalize its potential impacts on specific determinants of health.

By combining positive and adverse impacts of UA on the determinants of health, this review takes a holistic approach to invite practitioner, and policy makers to address UA challenges while promoting it. The insights gained from this study will encourage practitioners to test the urban soils prior to growing UA produce.

## Conclusion

This study illustrates a global picture of the current academic literature on the impacts of UA on the determinants of health. The study also designs the paths for future research in public health and urban planning domains. The inconsistency and the lack of strong quality in the methodology of the included studies are proof that more rigorous studies are needed to demonstrate the positive and adverse impacts of UA on different determinants of health. Nevertheless, the substantial existing evidence from this review corroborate that UA can influence different determinants of health such as food security, social capital, health and well-being in a variety of contexts (high-, middle-, low- income countries). In addition, UA practitioners can be motivated by social benefits such as supplying quality food and building social capital. There are also many physical and mental health benefits to different population groups. In a holistic sense, the evidence suggests benefits of UA on multiple dimensions of health with few adverse effects; thus, UA can be recommended as an intervention that positively influence the determinants of health. Concerns regarding urban soil contamination have to be addressed by analyzing physical and chemical proprieties of the soil and applying decontamination techniques when needed to ensure that there are no health risks to UA users.

Finally, we advocate for greater impact assessments by including transnational and multi--city approaches to compare the findings in different countries’ income level and geographic contexts. We also need a unified language to deal with heterogeneity in different types of UA identified.

## Additional files

Additional file 1: Full electronic search strategy for PubMed. (PDF 30 kb)

Additional file 2: Quality Appraisal for Quantitative studies and mixed methods studies. (XLSX 21 kb)

Additional file 3: Quality appraisal for qualitative and mixed methods studies. (XLSX 19 kb)

Additional file 4: Description of included studies. (XLSX 53 kb)

## Abbreviations

BMI: Body mass index
F&V: Fruit and vegetables
PICOS: Participants, Intervention, Context, Outcomes, and Study design
UA: Urban agriculture
