# Supplementary material for: Scoping review of the impacts of urban agriculture on the determinants of health
Source: BMC Public Health. 2019 May 31;19:672. doi: 10.1186/s12889-019-6885-z (PMC6545001; doi:10.1186/s12889-019-6885-z)
Supplement: Supplementary file 1 — Full electronic search strategy for PubMed. (PDF 30 kb) [file 12889_2019_6885_MOESM1_ESM.pdf]

PubMed search strategy performed: January 22, 2018

"Food Supply"[Mesh] OR "Food Security"[tiab] OR "Food Insecurity"[tiab] OR "Food Access"[tiab] OR "Food Availability"[tiab] OR "Food Quality"[Mesh:NoExp] OR "Food Quality"[tiab] OR "Food Safety"[Mesh:NoExp] OR "Food Safety"[tiab] OR "Food Contamination"[Mesh:NoExp] OR "Food"[Mesh:NoExp] OR "Health* Food"[tiab] OR "Income"[Mesh:NoExp] OR "Income"[tiab] OR "Cost Savings"[Mesh:NoExp] OR "Cost Savings"[tiab] OR "Poverty alleviation"[tiab] OR "Nutritional Status"[Mesh:NoExp] OR "Nutritional Status"[tiab] OR "Nutrient deficiency"[tiab] OR "Fruit and vegetable intake"[tiab] OR "Fruit and vegetable consumption"[tiab] OR "fruits and vegetables"[tiab] OR "Vegetable? intake"[tiab] OR "Vegetables"[Mesh:NoExp] OR "Fruit"[Mesh:NoExp] OR "Fruit? intake"[tiab] OR "Dietary diversity"[tiab] OR "Diet"[tiab] OR "Diet"[Mesh:NoExp] OR "Malnutrition"[Mesh:NoExp] OR "Malnutrition"[tiab] OR "Undernutrition"[tiab] OR "Overweight"[Mesh:NoExp] OR "Overweight"[tiab] OR "Obesity"[Mesh:NoExp] OR "Obesity"[tiab] OR "Quality of Life"[Mesh:NoExp] OR "Healthy Lifestyle"[Mesh:NoExp] OR "Healthy Lifestyle"[tiab] OR "Exercise"[Mesh:NoExp] OR "Physical activity"[tiab] OR "Leisure Activities"[Mesh:NoExp] OR "Leisure"[tiab] OR "Well-being"[tiab] OR "Interpersonal Relations"[Mesh:NoExp] "Interpersonal Relations"[tiab] OR "Social capital"[tiab] OR "Personal Development"[tiab] OR "Empowerment"[tiab] OR "education"[Mesh:NoExp] OR "nutrition education"[tiab] OR "Civic engagement"[tiab] OR "Community engagement"[tiab]OR "Mental Health"[Mesh:NoExp] OR "Mental Health"[tiab] OR "Dementia"[Mesh:NoExp] OR "Dementia"[tiab] OR "Stress, Psychological"[Mesh:NoExp] OR "stress"[tiab] OR "Perceptions of life"[tiab] OR "Cultural connection"[tiab] OR "Violence"[Mesh:NoExp] OR "Depression"[Mesh:NoExp] OR "Security perception"[tiab] OR "Health risk"[tiab] OR "Resilience"[tiab] OR "Pain"[Mesh:NoExp] OR "pain"[tiab] OR "Horticultural Therapy"[Mesh] OR "Therapeutic garden"[tiab]

AND

"Agriculture"[Mesh:NoExp] OR Agricultur*[tiab] OR "Food Production"[tiab] OR "Gardening"[Mesh] OR Community Garden*[tiab] OR Collective Garden*[tiab] OR Gardens[Mesh] OR Garden*[tiab] OR "Farms"[Mesh] OR Farm*[tiab] OR "Farms"[Mesh:NoExp] OR Allotment$[tiab] OR Horticultur*[tiab] OR Home garden*[tiab] OR Home garden*[tiab] OR "Aquaculture"[Mesh:NoExp] OR "Aquaculture"[tiab]

AND

"Cities"[Mesh:NoExp] OR City[tiab] OR Urban[tiab] OR Metropol*[tiab] OR Suburb*[tiab] OR Town*[tiab] OR Allotment$[tiab] OR Rooftop$[tiab]
